# Effect of Eccentric Cycling on Oxygen Uptake and Hemodynamics in Patients With Pulmonary Vascular Disease: A Randomized Controlled Crossover Trial

**DOI:** 10.1016/j.chpulm.2024.100054

**Published:** 2024-03-29

**Authors:** Julian Müller, Simon R. Schneider, Anna Titz, Claudia Thalmann, Esther I. Schwarz, Christoph Bauer, Ekkehard Grünig, Malcolm Kohler, Mona Lichtblau, Silvia Ulrich

**Affiliations:** a Department of Pulmonology, University Hospital Zurich, Zurich, Switzerland; b University of Zurich, Zurich, Switzerland; c Faculty of Sport and Health Science, University of Jyväskylä, Jyväskylä, Finland; d Lake Lucerne Institute, Vitznai, Switzerland; e Thoraxclinic Heidelberg, Heidelberg, Germany

**Keywords:** cardiopulmonary exercise testing, CTEPH, eccentric cycling exercise, PAH, pulmonary hypertension, pulmonary vascular disease, rehabilitation

## Abstract

**Background:**

Eccentric cycling exercise (ECC) allows training at low metabolic costs and may therefore be valuable for patients with precapillary pulmonary hypertension (PH) due to pulmonary vascular disease (PVD).

**Research Question:**

What are the ventilatory and circulatory responses of ECC vs concentric cycling exercise (CON) in patients with PVD?

**Study Design and Methods:**

This was a randomized controlled crossover trial in which patients diagnosed with PVD, defined as either pulmonary arterial or chronic thromboembolic PH, performed CON and ECC cycling tests at identical submaximal work rates, following stepwise incremental protocols. Oxygen uptake and additional cardiorespiratory responses were measured breath-by-breath by ergospirometry. Hemodynamic parameters (eg, systolic pulmonary arterial pressure [sPAP], tricuspid annular plain systolic excursion) were measured by echocardiography.

**Results:**

Thirty-three patients (19 with pulmonary arterial hypertension and 14 with chronic thromboembolic PH; 13 female; mean age, 50 ± 15 years) were included. At identical work rates during ECC compared with CON, oxygen uptake was significantly lower by −200 mL/min (−40%; 95% CI, −272 to −129; *P* < .01), minute ventilation was significantly lower by −5.5 L/min (−30%; 95% CI, −9.2 to −3.1; *P* < .01), and sPAP was significantly lower by −12 mm Hg (−20%; 95% CI, −20 to −4; *P* < .01). Right ventricular-arterial coupling, as measured by tricuspid annular plain systolic excursion/sPAP, was 0.11 mm/mm Hg higher (31%; 95% CI, 0.04-0.18; *P* < .01). No adverse events occurred.

**Interpretation:**

This study supports the hypothesis that ECC is a feasible and well-tolerated exercise modality for patients with PVD, with lower oxygen demand and a reduced load on the right ventricle. Future studies should investigate whether ECC improves exercise capacity, muscle force, and possibly hemodynamics during prolonged rehabilitation programs in patients with PVD.

**Clinical Trial Registration:**

ClinicalTrials.gov; No.: NCT05186987; URL: www.clinicaltrials.gov

**Take-home Points**

**Study Question:** This trial investigated the ventilatory and circulatory differences between eccentric and ordinary concentric cycling exercise in 33 patients with pulmonary arterial or chronic thromboembolic pulmonary hypertension, collectively categorized as pulmonary vascular disease.

**Results:** Oxygen uptake and pulmonary arterial pressure were both significantly lower during eccentric compared with concentric cycling. At the same time, right ventricular-arterial coupling was increased by 30% during eccentric cycling.

**Interpretation:** Eccentric cycling was shown to be a feasible and well-tolerated exercise modality for patients with pulmonary vascular disease, resulting in lower oxygen demand, ventilation, and dyspnea perception. This is combined with a lower right ventricular load, as expressed in a better contractile response to increased afterload during eccentric compared with concentric cycling.

During eccentric cycling exercise (ECC) training, the cyclist has to resist the backward movement of the pedals on a special motor-driven ergometer. The load on the pedals exceeds the torque generated by the lengthening muscle (negative work), generating and storing elastic recoil energy.^1^, ^2^, ^3^ ECC generates high forces but requires up to 80% less oxygen^4^ than conventional concentric cycling exercise (CON). Therefore, it is described as a promising and potentially effective training modality with low metabolic costs.^1^^,^^5^^,^^6^ Prolonged and regular exercise training leads to chronic adaptations. Recently, ECC has been found to be more effective than CON because it increases muscle strength, hypertrophy, 6-min walking distance, and, notably, maximum oxygen uptake ($\dot{V}$o_2_), especially in patients with COPD, chronic left heart failure, or coronary heart disease.^7^ Furthermore, there is evidence for hemodynamic specificities (ie, lower cardiac output [CO]) during ECC at the same work rate.^8^ Accordingly, ECC could be a promising addition to pharmaceutical treatment for patients with precapillary pulmonary hypertension (PH) due to pulmonary vascular disease (PVD) (eg, pulmonary arterial hypertension [PAH], chronic thromboembolic pulmonary hypertension [CTEPH]). Pharmaceutical and interventional treatments have improved various outcomes for patients with PVD, but many of them still experience exertional dyspnea associated with reduced exercise capacity and quality of life.^9^^,^^10^ Therefore, evidence-based and innovative therapies are needed.^11^

Several randomized controlled trials and meta-analyses have shown that exercise training in patients with PVD increases 6-min walking distance, peak $\dot{V}$o_2_, peak workload, and quality of life.^12^, ^13^, ^14^, ^15^, ^16^ Subsequently, the European Respiratory Society Task Force^11^ published a review on the efficacy, cost-effectiveness, and safety of exercise training and rehabilitation in patients with PVD in 2019. In addition, a large European multicenter study in 2021 showed that supervised exercise training is safe and well-tolerated in patients with PVD.^17^ These publications were instrumental in obtaining a 1A recommendation for supervised exercise training for patients with stable PH in the guidelines.^9^

Nevertheless, exercise intensities are low in most patients with advanced PVD, and the major cause of limitation is cardiovascular rather than ventilatory or muscular.^18^ There are still concerns that the higher blood flow during exercise may increase pulmonary vascular remodeling and accelerate disease progression in patients with PVD. Therefore, low-intensity exercise protocols are recommended.^11^ Despite this, many patients with PVD are almost physically unable to perform exercise at a beneficial level. Thus, ECC may be a promising exercise modality, especially for this highly vulnerable cohort, allowing comparatively high work rates combined with low metabolic demands and less circulatory strain.^19^

The aim of this study was to compare the ventilatory and circulatory responses at identical submaximal loads for ECC vs CON in patients with PVD to provide a basis for future longer-term training and rehabilitation studies.

## Study Design and Methods

This was a randomized controlled crossover trial conducted from December 2022 to August 2023. Participants were recruited during PVD consultations from October 2022 to July 2023. Randomization was performed using software-based block randomization with randomly computed block lengths. Allocation to sequences was concealed and performed by researchers from the department. After data collection, participants’ data were anonymized to prevent access to source data. Patients with stable PVD of all sexes between 18 and 80 years of age who were diagnosed with either PAH or distal CTEPH, according to current guidelines,^9^ and who had been on unchanged medication for > 4 weeks were included. Patients with severe daytime hypoxemia (Pao_2_ < 7.3 kPa) were excluded.

All patients gave their written informed consent for participation and further use of their data for scientific analyses. The study is in accordance with the Declaration of Helsinki, has been approved by the local ethical authorities (KEK 2021-0132), and is registered on clinicaltrials.gov (NCT05186987).

### Cycling Exercise

Interventions were performed on two different days to avoid carryover effects. Prior to the first experimental session, a familiarization session on the eccentric ergometer took place to give patients the opportunity to learn how to exert force on the pedals accurately and to avoid muscle soreness. Participants performed two submaximal, standardized, identical stepwise incremental cycling exercise tests with cycling intervals of 3 to 5 min per step (9-15 min total) at pedaling rates of 55 to 65 revolutions per minute (rpm): one with a CON (Ergoselect 200; Ergoline GmbH) and one with an ECC (Cyclus 2 Recumbent; RBM elektronik-automation GmbH) in a randomized order. The intensity started at 20 to 50 W and increased by 10 to 20 W per step, depending on participants’ fitness levels. The patients were connected via a mouthpiece (Ergostick; Geratherm Medical) to the flow sensor of a metabolic unit, which was calibrated before each test and measured respiratory gas exchange. $\dot{V}$o_2_ and additional cardiorespiratory responses (tidal volume [VT], breathing frequency, minute ventilation [$\dot{V}$e], CO_2_ output [$\dot{V}$co_2_], respiratory exchange ratio, and derived variables) were recorded breath by breath. The physiologic dead space fraction was computed from arterial and end-tidal Pco_2_.^20^ Arterial oxygen saturation was measured continuously by finger clip pulse oximetry. $\dot{V}$o_2_ efficiency slope was calculated as regression slope *a* in $\dot{V}$o_2_ = *a* log $\dot{V}$e + *b*.^21^

Echocardiography was performed at rest as a baseline measurement and both before and during exercise within the last minute of each step. Echocardiographic recordings were conducted using a real-time sector scanner (CX 50; Philips) with an integrated color, continuous wave, and pulsed wave Doppler system. Recordings and measurements were performed according to guidelines of the American Society of Echocardiography.^22^ To estimate systolic pulmonary arterial pressure (sPAP), the maximal pressure gradient of tricuspid regurgitation was calculated from maximal tricuspid regurgitation velocity determined with continuous wave Doppler using the modified Bernoulli equation, ΔPressure = 4 × TRV_max_^2^, where TRV is tricuspid regurgitation velocity, without adding the right atrial pressure. sPAP/CO_slope_ was calculated as follows: (sPAP_peak exercise_ − sPAP_rest_)/(CO_peak exercise_ − CO_rest_).

Heart rate was derived from a 12-lead ECG, and BP was measured using automated arm cuff measurements. At rest and during the last 30 s of exercise, arterial blood samples were taken from the radial artery for immediate analysis of arterial blood gases (ABL90 FLEX; Radiometer). After each test, participant-reported outcomes, Borg CR10 scale for perceived leg fatigue, and dyspnea were assessed.

### Sample Size, Data Presentation, and Statistics

Based on the primary outcome $\dot{V}$o_2_, a sample size of 28 was calculated, assuming a minimal clinically important difference (MCID) of 30 ± 30 mL/min^23^ with a significance level of 0.05 and power of 0.9. To account for dropouts, we aimed to include 32 participants. To compare the main outcome between concentric and eccentric cycling at the end of each step, physiologic values were averaged and compared over the last 30 s of the three steps. Data were summarized as mean ± SD. A linear mixed model was fitted to the data using intervention (CON vs ECC), period, and intervention-period interaction as fixed effects and patient as random intercept; therefore, carryover effects (treatment-period interaction) and period effects were controlled according to the standards of crossover trials. We tested whether the intervention-period interaction could be removed from the model. Model assumptions (eg, normal distribution) were checked by visual inspection of the homogeneity and normality of the residuals and random effects.

The analysis of the secondary outcomes followed the same procedure as previously described, with the addition of baseline characteristics as covariates to minimize bias. The linear mixed model dealt with missing data,^24^ and an intention-to-treat analysis was used.

In all analyses, a 95% CI that excluded the null effect was considered as evidence of statistical significance. Analyses were performed using RStudio software version 4.1.0 (Posit PBC).

## Results

Model assumptions of homogeneity and normality of the residuals and random effects were met, and there were neither carryover nor period effects.

**Figure 1:**
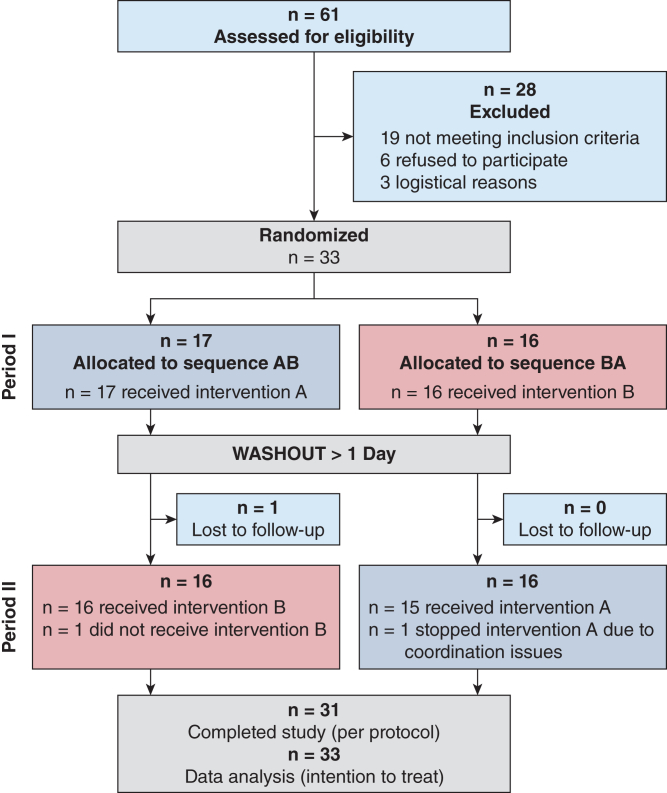
Patient flow for crossover trials according to the Consolidated Standards of Reporting Trials statement for crossover trials.^25^

A total of 33 participants (19 with PAH and 14 with CTEPH; 13 female; mean age, 50 ± 15 years) were included, of whom 31 completed both periods of the trial. One participant dropped out of the eccentric cycling test due to coordination issues, and one participant was unable to participate in period 2 (concentric cycling) for personal reasons. For these two incomplete data sets, only one period was analyzed according to an intention-to-treat approach. Baseline characteristics of the participants are listed in Table 1. The flowchart of the study is shown in Figure 1.^25^

**Table 1:** Patient Characteristics (N = 33)

Baseline Characteristics	Value
Female/male	13 (39)/20 (61)
Age, y	50 ± 15
Pulmonary arterial hypertension	19 (58)
Idiopathic	11 (58)
Heritable	2 (11)
Associated with:	
Congenital heart disease	3 (16)
Connective tissue disease	1 (5)
Portopulmonary hypertension	1 (5)
Veno-occlusive disease	1 (5)
Chronic thromboembolic pulmonary hypertension	14 (42)
Persistent after pulmonary endarterectomy	5 (15)
New York Heart Association functional class, I-III	I: 11 (33), II: 15 (46), III: 7 (21)
BMI, kg/m^2^	26.7 ± 4.7
Spo_2_ at rest, %	94 ± 3
NT-proBNP, ng/L	244 ± 281
Maximum work rate, W	136 ± 58
Peak oxygen uptake, mL/kg/min	18.8 ± 6.9
6-min walking distance, m	582 ± 98
Echocardiography at rest	
Left ventricular ejection fraction, %	58 ± 6
Tricuspid regurgitation velocity, cm/s	278.2 ± 98.4
Systolic pulmonary artery pressure, mm Hg	42.5 ± 17.1
Tricuspid annular plane systolic excursion, mm	17.6 ± 3.8
Tissue Doppler index s', mm/s	11.8 ± 2.8
Pulmonary acceleration time, ms	99 ± 30
Fractional area change, %	37.4 ± 6.4
Pulmonary hypertension-targeted medication	
Endothelin receptor antagonist	23 (70)
Phosphodiesterase-5 inhibitor	9 (27)
Prostanoids	5 (15)
Selexipag	3 (9)
Riociguat	12 (36)
Calcium channel blocker	6 (18)
β-blockers	1 (3)

Data are presented as mean ± SD or No. (%). Values at peak exercise were taken from medical records. NT-proBNP = N-terminal pro b-type natriuretic peptide; Spo_2_ = Arterial oxygen saturation by pulse oximetry

### Ventilation, Gas Exchange, and Exertion

**Figure 2:**
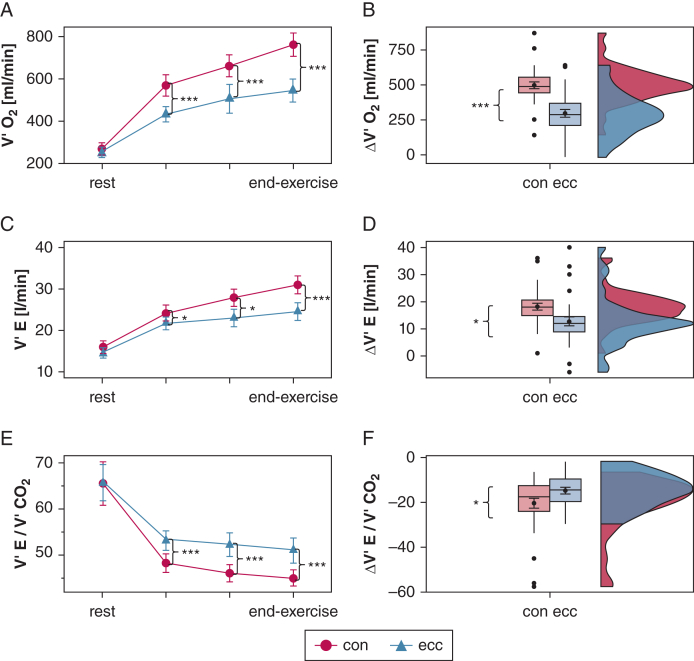
A-F, Ventilator parameters and gas exchange during eccentric (blue triangle) compared with traditional concentric (red circles) stepwise incremental cycling exercise in 33 patients with pulmonary vascular disease, according to a randomized crossover design at identical workloads. Data are presented as means with SDs and the corresponding statistically significant differences between the two conditions (∗*P* < .05; ∗∗∗*P* < .01). A, B, $\dot{V}$o_2_. A, Absolute difference between ecc and con during exercise. B, Changes from baseline (Δ) and the data distribution. C, D, $\dot{V}$e. C, Absolute difference between ecc and con during exercise. D, Changes from baseline (Δ) and the data distribution. E, F, $\dot{V}$e/$\dot{V}$co_2_. E, Absolute difference between ecc and con during exercise. F, Changes from baseline (Δ) and the data distribution. con = concentric cycling exercise; ecc = eccentric cycling exercise; $\dot{V}$co_2_ = CO_2_ output; $\dot{V}$e = minute ventilation; $\dot{V}$o_2_ = oxygen uptake.

Despite identical work rates at end exercise (CON vs ECC), $\dot{V}$o_2_ was 763 ± 159 (end exercise – rest [Δrest], 495) vs 545 ± 156 (Δrest, 295) mL/min, respectively. The mean difference was statistically significantly lower by −200 mL/min (95% CI, −272 to −129; *P* < .01) in ECC compared with CON (Fig 2, Table 2).

**Table 2:** Ergospirometry Data

Parameter	Rest		End Exercise (44 ± 10 W)		ΔEccentric-ΔConcentric	
	Concentric Mean ± SD	Eccentric Mean ± SD	Concentric Mean ± SD (Δrest)	Eccentric Mean ± SD (Δrest)	Mean Change (95% CI), % Change	*P* Value
$\dot{V}$o_2_, mL/min	268 ± 83	250 ± 69	763 ± 159 (495)	545 ± 156 (295)	−200 (−272 to −129), 40	**< .01**
$\dot{V}$co_2_, mL/min	243 ± 84	219 ± 68	717 ± 160 (474)	507 ± 193 (288)	−186 (−267 to −105), 39	**< .01**
$\dot{V}$e, L/min	15.3 ± 4.4	14.1 ± 3.5	33.6 ± 8.4 (18.3)	26.9 ± 9.6 (12.8)	−5.5 (−9.2 to −3.1), 30	**< .01**
VT, L	0.9 ± 0.4	0.7 ± 0.2	1.5 ± 0.5 (0.6)	1.1 ± 0.4 (0.4)	−0.2 (−0.3 to −0.1), 33	**.04**
Bf, L/min	17.6 ± 5.5	20.7 ± 5.0	24.0 ± 5.6 (6.4)	25.8 ± 6.6 (5.1)	−1.3 (−3.5 to 1.1), 20	.30
Pao_2_, kPa	10.4 ± 1.5		9.0 ± 2.2	9.8 ± 1.7	0.8 (−0.2 to 1.6), 9	.15
Paco_2_, kPa	4.7 ± 0.8		4.9 ± 0.4	4.6 ± 0.6	−0.3 (−0.6 to −0.1), 6	**.02**
Sao_2_, %	95.8 ± 1.7		93.0 ± 4.2	95.1 ± 2.9	2.1 (−0.5 to 4.6), 2	.07
Arterial pH	7.427 ± 0.1		7.418 ± 0.03	7.443 ± 0.03	0.025 (0.004 to 0.043), 0.3	**.03**
Lactate, mmol/L	1.1 ± 1.3		1.9 ± 1.0	1.5 ± 1.4	−0.4 (−1.2 to 0.4), 21	.38
Bicarbonate, mmol/L	24.1 ± 2.7		23.9 ± 1.8	24.4 ± 1.3	0.5 (−0.4 to 1.3), 2	.31

The work rate at end exercise was identical for eccentric cycling exercise and concentric cycling exercise. Δrest = end exercise – rest; ΔEccentric-ΔConcentric = delta (end exercise minus rest) eccentric minus delta contrentric; Bf = breathing frequency; Sao_2_ = arterial oxygen saturation; $\dot{V}$co_2_ = CO_2_ output; $\dot{V}$e = minute ventilation; $\dot{V}$o_2_ = oxygen uptake; VT = tidal volume.

At end exercise, $\dot{V}$e was 5.5 L/min (*P* = .01) lower, which was due to a reduction in VT and breathing frequency of −0.2 L (*P* = .04) and −1.2 breaths/min (*P* = .30), respectively, during ECC compared with CON (for all 95% CIs, see Table 2). The Paco_2_-end-tidal CO_2_ gradient and physiologic dead space fraction were both unchanged between ECC and CON. The $\dot{V}$e/$\dot{V}$co_2_ slope was higher by 3 (*P* = .02), whereas $\dot{V}$o_2_ efficiency slope was lower by −0.3 L/min (*P* < .01) during ECC compared with CON.

Borg CR10 for perceived dyspnea was 19% lower for ECC than CON (*P* = .02), and there was no statistically significant difference for perceived leg fatigue.

There was no statistically significantly difference in Pao_2_, arterial oxygen saturation, arterial lactate, and bicarbonate at end exercise between ECC and CON. Paco_2_ was −0.3 kPa lower (*P* = .02) in ECC compared with CON (for all 95% CIs, see Table 3).

**Table 3:** Ergospirometry and echocardiography at end exercise

End Excercise	Concentric	Eccentric	Mean Difference (95% CI), % Change	*P* Value
Ergospirometry data at end exercise (step 3, 44 ± 10 W)				
% of peak $\dot{V}$o_2_	56 ± 16	37 ± 13	…	…
$\dot{V}$o_2_, mL/kg/min	9.7 ± 2.2	7.0 ± 2.3	−2.7 (−3.4 to −1.9), 28	< .01
$\dot{V}$o_2_/WR, mL/min/W	11.5 ± 2.6	6.9 ± 3.6	−4.6 (−5.8 to −3.2), 40	< .01
$\dot{V}$e/$\dot{V}$o_2_	42.4 ± 6.5	46.4 ± 8.2	4.0 (2.1 to 6.4), 9	< .01
$\dot{V}$e/$\dot{V}$co_2_	44.9 ± 4.9	50.9 ± 7.9	6.0 (3.8 to 8.5), 13	< .01
$\dot{V}$e/$\dot{V}$co_2 slope_	36.0 ± 5.7	39.0 ± 9.1	3.0 (0.5 to 5.4), 8	.02
Paco_2_-end-tidal CO_2_, kPa	2.22 ± 0.4	2.19 ± 0.6	0.03 (−0.04 to 0.03)	.91
Dead space fraction	0.45 ± 0.06	0.47 ± 0.09	0.02 (−0.04 to 0.02)	.13
RER	0.9 ± 0.1	0.9 ± 0.1	0 (−0.1 to 0.0), 0	.05
Spo_2_, %	92 ± 3	92 ± 3	0 (−0.8 to 1), 1	.66
Oxygen uptake efficiency slope, L/min	1.4 ± 0.4	1.1 ± 0.4	−0.3 (−0.4 to −0.1), 21	< .01
Borg CR10 perceived dyspnea	3.2 ± 1.9	2.6 ± 1.9	−0.6 (−0.9 to −0.1), 19	.02
Borg CR10 perceived leg fatigue	3.2 ± 1.6	3.6 ± 2.4	0.4 (−0.4 to 1.3), 13	.30
Circulatory data at end exercise (step 3, 44 ± 10 W)				
TRVmax, ms	376 ± 69	331 ± 77	−45 (−71 to −16), 12	< .01
sPAP, mm Hg	59 ± 21	47 ± 22	−12 (−20 to −4), 20	< .01
Heart rate, beats/min	109 ± 16	102 ± 18	−7 (−11 to −2), 6	.01
VTI, cm	21.4 ± 3.9	21.2 ± 3.4	−0.2 (−1.5 to 1.5), 1	.79
Stroke volume, mL	70 ± 25	67 ± 14	−3 (−8 to 3), 4	.38
Cardiac output, L/min	7.4 ± 2.4	6.7 ± 1.7	−0.7 (−1.4 to 0.1), 10	.08
sPAP/CO_slope_, mm Hg/L/min	12.1 ± 10.8	10.0 ± 6.8	−2.1 (−11.5 to 7.3), 17	.64
TAPSE, mm	19.4 ± 4.0	20.1 ± 4.2	0.7 (−0.5 to 1.9), 3	.23
TAPSE/sPAP, mm/mm Hg	0.36 ± 0.1	0.47 ± 0.2	0.11 (0.04 to 0.18), 31	< .01
Systolic BP, mm Hg	131 ± 16	136 ± 22	5 (−4 to 15), 4	.25
Diastolic BP, mm Hg	70 ± 11	80 ± 10	10 (6 to 15), 14	< .01

Data are presented as mean ± SD or as otherwise indicated. The work rate at end exercise was identical for eccentric cycling exercise and concentric cycling exercise. CO = cardiac output; RER = respiratory exchange ratio; sPAP = systolic pulmonary arterial pressure; Spo_2_ = oxygen saturation by pulse oximetry; TAPSE = tricuspid annular plane systolic excursion; TRVmax = maximum tricuspid regurgitation velocity; $\dot{V}$co_2_ = CO_2_ output; $\dot{V}$e = minute ventilation; $\dot{V}$o_2_ = oxygen uptake; VTI = velocity time integral; WR = work rate.

### Circulation and Right Heart Function

During exercise, sPAP was significantly lower by −12 mm Hg (95% CI, −20 to −4; *P* < .01) at end exercise in ECC compared with CON.

**Figure 3:**
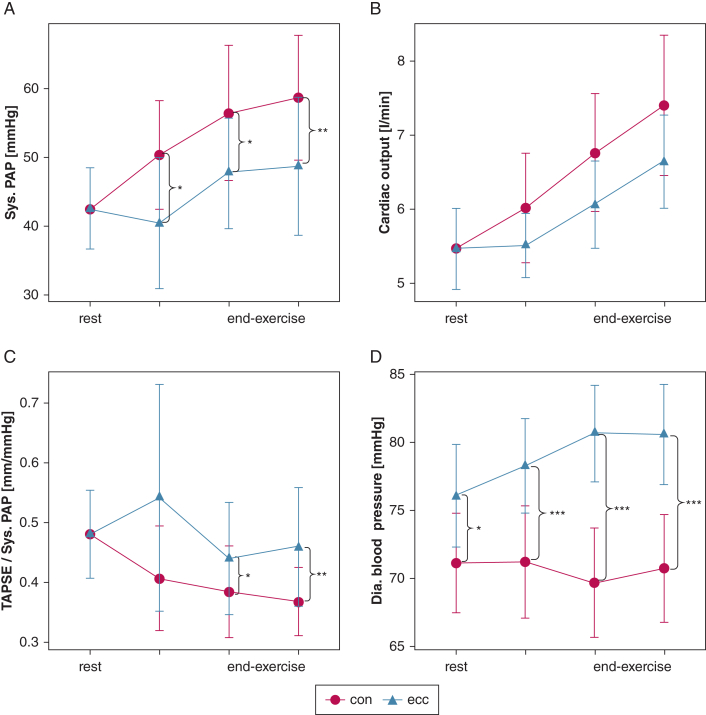
A-D, Circulatory parameters during eccentric (blue triangle) compared with traditional concentric (red circles) stepwise incremental cycling exercise in 33 patients with pulmonary vascular disease, according to a randomized crossover design at identical workloads. Data are presented as means with SDs and the corresponding statistically significant differences between the two conditions (∗*P* < .05; ∗∗*P* = .01; ∗∗∗*P* <.01): (A) Sys. PAP, (B) cardiac output, (C) right ventricular-arterial coupling expressed in TAPSE/Sys. PAP, and (D) diastolic blood pressure. con = concentric cycling exercise; Dia. = diastolic; ecc = eccentric cycling exercise; Sys. PAP = systolic pulmonary artery pressure; TAPSE = tricuspid annular plane systolic excursion.

At end exercise, heart rate was lower by −7 beats/min in ECC compared with CON (*P* = .01), whereas stroke volume, CO, sPAP/CO, sPAP/CO_slope_, and tricuspid annular plane systolic excursion (TAPSE) remained unchanged between ECC and CON. TAPSE/sPAP was 31% higher in ECC at end exercise compared with CON (*P* < .01). Diastolic BP was up to 10 mm Hg higher during ECC compared with CON (*P* < .01) (for all 95% CIs, see Fig 3 and Table 3).

## Discussion

To our knowledge, this was the first randomized controlled crossover trial to examine the differences in $\dot{V}$o_2_ and other ventilatory and circulatory responses between ECC and CON in 33 patients with PVD. $\dot{V}$o_2_ was significantly lower in ECC compared with CON at end exercise. This is clinically relevant and is reflected in the significantly lower perception of dyspnea.

### Ventilation, Gas Exchange, and Rating of Exertion

In this study comparing submaximal ECC vs CON in patients with PVD, we found that ECC was associated with a decreased $\dot{V}$o_2_ from the beginning to end exercise. The lower $\dot{V}$o_2_ during ECC may be clinically relevant for these patients; however, the MCID is determined for peak $\dot{V}$o_2_ (not for submaximal $\dot{V}$o_2_). The reported MCID is 1 to 1.5 mL/kg/min or 104 mL/min, determined by the improvements observed after pulmonary rehabilitation. Nevertheless, the lower $\dot{V}$o_2_ during ECC remains above this threshold.^26^^,^^27^ Cycling intensity in our trial was set submaximally to reflect intensities considered safe for training in patients with PVD,^17^ reaching 44 ± 10 W at step 3. This corresponded to 56% of peak $\dot{V}$o_2_ in CON and 33% in ECC. Despite identical work rates, the cardiopulmonary intensity at end exercise was almost halved during ECC compared with CON. This was also emphasized by the significantly reduced $\dot{V}$o_2_/work rate during ECC. Other trials in healthy individuals and in patients with COPD, coronary artery disease, or chronic heart failure have shown that after several familiarization sessions and at higher work rates, the difference in $\dot{V}$o_2_ between CON and ECC can be up to 50% in patients and 70% in healthy individuals.^1^^,^^4^^,^^28^ In the current trial, participants received only one familiarization session with the aim of reducing the risk of muscle soreness and damage, according to the repeated bout effect.^29^ Therefore, there is a high likelihood that patients with PVD would also be able to economize and optimize ECC by further increasing the difference in $\dot{V}$o_2_, to exercise on comparable high work rates with only a minimum $\dot{V}$o_2_.

Ventilation, expressed as $\dot{V}$e, was lower during ECC than CON at the same work rate, most probably due to lower oxygen demand and CO_2_ production. Because patients with PVD typically hyperventilate at rest and during exercise due to increased chemosensitivity,^30^ dyspnea perception, and dead space ventilation, which contributes to exercise limitation, the lower $\dot{V}$e during ECC might be beneficial for patients with PVD.^18^^,^^31^ However, Paco_2_-end-tidal CO_2_ gradient and dead space fraction were unchanged at end exercise. Of interest, both $\dot{V}$e/$\dot{V}$co_2_ and $\dot{V}$e/$\dot{V}$co_2 slope_ were significantly increased during ECC. During CON, breathing patterns are physiologically economized with increasing metabolic demand and ventilatory drive.^32^ Hence, for submaximal protocols combined with lower oxygen demand and CO_2_ production during ECC, the increased $\dot{V}$e/$\dot{V}$co_2_ and $\dot{V}$e/$\dot{V}$co_2 slope_ may indicate that there was less need to economize breathing patterns during ECC compared with CON, and these parameters appear to be rather not clinically relevant.

$\dot{V}$o_2_ efficiency slope, a concept aimed at providing a submaximal measure of cardiopulmonary functional reserve that correlates with peak $\dot{V}$o_2_, was 1.4 during CON and statistically significantly lower in ECC.^33^ The $\dot{V}$o_2_ efficiency slope could be of potential value for predicting peak $\dot{V}$o_2_ and determining exercise intensity during pulmonary rehabilitation without the need for exercise testing to exhaustion.^34^

Arterial blood gas analysis at end exercise showed that Pao_2_ and arterial oxygen saturation were slightly, but not statistically significantly, higher, whereas Paco_2_ was lower in ECC, which is in line with the ventilatory findings. We found that arterial lactate increased at submaximal exercise in both and was slightly, but not statistically significantly, lower in ECC compared with CON. Arterial pH was higher in ECC due to lower Paco_2_.

An important finding for patients with PVD is the significantly lower perceived dyspnea of −19% at end exercise during ECC. Perceived leg fatigue, however, was not significantly different. This may suggest that participants tolerated submaximal ECC well and that there was no acute muscle soreness. However, the latter may occur later. In line with our results, Nickel et al^35^ exposed 10 patients with COPD to high-intensity ECC vs CON and reported reductions in $\dot{V}$o_2_ by −52%, $\dot{V}$e by −47%, and perceived dyspnea by −51% in ECC.

### Circulation and Right Heart Function

The increase in sPAP was significantly lower in ECC compared with CON. This points toward a potential training opportunity with lower afterload to the right ventricle. Furthermore, a lower sPAP at rest^36^ and during exercise^37^^,^^38^ is of prognostic relevance in patients with PVD; however, the latter was always assessed during CON. Nevertheless, a lower sPAP is associated with lower right ventricular load and a lower risk of decompensation during exercise.^9^ This is further supported by the higher TAPSE/sPAP during ECC, an echocardiographic parameter that assesses the right ventricular contractile response to increased afterload, also known as right ventricular-arterial coupling.^39^^,^^40^ When the adaptation of the right ventricle to preserve its arterial coupling is exhausted, this leads to dilatation and right-sided heart failure.^18^ The CO and sPAP/CO slope were only slightly, but not statistically significantly, lower in ECC compared with CON. However, it is known that there are limitations in assessing CO by echocardiography that lead to rather large SDs. We hypothesize that due to the lower metabolic demand during ECC, there might be lower preload and afterload conditions during exercise. The right ventricle could then effortlessly preserve its coupling to the pulmonary circulation, resulting in less right side of the heart strain during ECC compared with CON.

To our knowledge, there has been only one study in which hemodynamics were measured invasively by right heart catheterization during ECC vs CON in 13 patients with coronary artery disease. This study reported lower pulmonary capillary wedge pressure, lower cardiac index, and slightly higher BP during ECC, despite fourfold higher work rates in ECC than in CON.^41^

The patients in our trial were stable and treated with PH-targeted medication and were mainly in functional class I and II. Thus, our results may not be applicable to more severely limited patients with PVD, a group that is also referred for pulmonary rehabilitation. Future studies investigating long-term eccentric training should also include more severely impaired patients.

Although several modes of exercise and rehabilitation programs for patients with PVD have been published, evidence for ECC is scarce.^42^ To our knowledge, this is the first randomized controlled trial that investigated ECC in these patients.

A recently published systematic review with meta-analysis demonstrated the superiority of ECC training compared with CON training by increasing muscle strength, hypertrophy, and maximum $\dot{V}$o_2_, especially in patients with COPD, chronic heart failure, or coronary artery disease. Additionally, ECC has been found to increase functional outcomes (eg, 6-min walking distance).^7^

Therefore, ECC seems to perfectly meet the needs of patients with PVD. The next step will therefore be to investigate whether ECC offers similar benefits for patients with PVD as have been described for other cardiopulmonary diseases.

**Figure 4:**
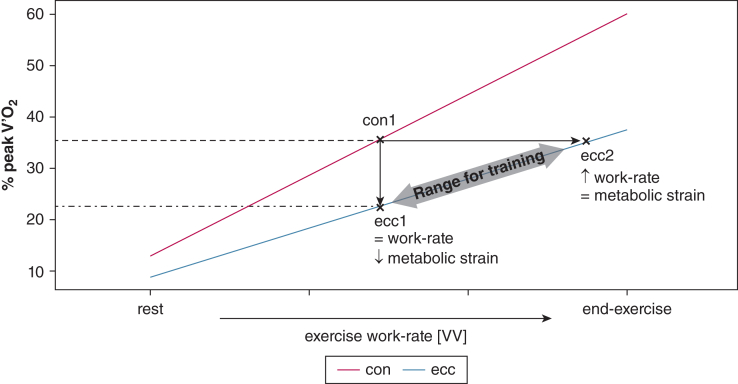
Plotted regression slope model with the slope of % $\dot{V}$o_2_ during ecc (blue) vs con (red). This is a theoretical illustration of potential exercise intensities. From the % $\dot{V}$o_2_, the aimed con exercise intensity can be derived. From the con slope, two ecc points can be inferred, ecc1 with the same work rate as con but with lower metabolic strain, and ecc2 with increased work rate combined with similar metabolic strain. The training intensity could lie between ecc1 and ecc2, and we would recommend starting with ecc1 and ramping it toward ecc2 in patients with pulmonary vascular disease. con = concentric cycling exercise; ecc = eccentric cycling exercise; $\dot{V}$o_2_ = oxygen uptake.

A potential upcoming challenge for ECC training in PVD will be the choice of appropriate intensity. In our experience, how well individual patients cope with ECC is multifactorial and does not necessarily correlate with level of fitness. Key factors include regular exposure to eccentric muscle work (eg, hiking downhill, walking downstairs, skiing in the past). We have created a graphical model that might help determine ECC intensity (Fig 4).

A limitation of the study could be that the slightly different body position of ECC and CON might have influenced the results. Although CON was performed upright, ECC was performed in Fowler’s position for safety reasons. However, we think that the hemodynamic difference between upright and Fowler, with the upper body being almost upright, can be judged as marginal. Because we included both patients with PAH and CTEPH, there might be some degree of heterogeneity according to the different PVD etiologies. However, there were no significant differences between patients with PAH and CTEPH. Furthermore, we only showed the acute response to ECC compared with CON. The effect of ECC in PVD in the long-term course needs further investigation.

## Interpretation

The current randomized controlled trial supports the hypothesis that ECC is a feasible and well-tolerated exercise modality for patients with PVD. ECC is associated with lower oxygen demand, ventilation, and dyspnea perception, combined with a lower right ventricular load, as reflected by increased ventricular-arterial coupling during ECC compared with CON. Hence, these findings call for a pulmonary rehabilitation study that includes patients with PVD and uses ECC to determine whether ECC holds promise as a highly effective training tool.

## Funding/Support

This study was financially supported by a grant from Janssen-Cilag GmbH.

## Financial/Nonfinancial Disclosures

The authors have reported to *CHEST Pulmonary* the following: E. G. has received fees for lectures and/or consultations from Actelion, Bayer/MSD, Ferrer, GEBRO, GSK, Janssen, and OMT; and research grants to his institution have been received from Acceleron, Actelion, BayerHealthCare, MSD, Bellerophon, GossamerBio, GSK, Janssen, Novartis, OMT, Pfizer, REATE, and United Therapeutics. None declared (J. M., S. R. S., A. T., C. T., E. I. S., C. B., M. K., M. L., S. U.).

## References

[1] Barreto R.V., de Lima L.C.R., Denadai B.S. (2021). Moving forward with backward pedaling: a review on eccentric cycling. Eur J Appl Physiol.

[2] Isner-Horobeti M.E., Dufour S.P., Vautravers P., Geny B., Coudeyre E., Richard R. (2013). Eccentric exercise training: modalities, applications and perspectives. Sports Med.

[3] Lindstedt S.L., LaStayo P.C., Reich T.E. (2001). When active muscles lengthen: properties and consequences of eccentric contractions. News Physiol Sci.

[4] Perrey S., Betik A., Candau R., Rouillon J.D., Hughson R.L. (2001). Comparison of oxygen uptake kinetics during concentric and eccentric cycle exercise. J Appl Physiol (1985).

[5] LaStayo P.C., Pierotti D.J., Pifer J., Hoppeler H., Lindstedt S.L. (2000). Eccentric ergometry: increases in locomotor muscle size and strength at low training intensities. Am J Physiol Regul Integr Comp Physiol.

[6] Roig M., O’Brien K., Kirk G. (2009). The effects of eccentric versus concentric resistance training on muscle strength and mass in healthy adults: a systematic review with meta-analysis. Br J Sports Med.

[7] Barreto R.V., de Lima L.C.R., Borszcz F.K., de Lucas R.D., Denadai B.S. (2023). Chronic adaptations to eccentric cycling training: a systematic review and meta-analysis. Int J Environ Res Public Health.

[8] Dufour S.P., Lampert E., Doutreleau S. (2004). Eccentric cycle exercise: training application of specific circulatory adjustments. Med Sci Sports Exerc.

[9] Humbert M., Kovacs G., Hoeper M.M. (2022). 2022 ESC/ERS guidelines for the diagnosis and treatment of pulmonary hypertension. Eur Heart J.

[10] Hoeper M.M., Huscher D., Ghofrani H.A. (2013). Elderly patients diagnosed with idiopathic pulmonary arterial hypertension: results from the COMPERA registry. Int J Cardiol.

[11] Grünig E., Eichstaedt C., Barberà J.-A. (2019). ERS statement on exercise training and rehabilitation in patients with severe chronic pulmonary hypertension. Eur Respir J.

[12] Mereles D., Ehlken N., Kreuscher S. (2006). Exercise and respiratory training improve exercise capacity and quality of life in patients with severe chronic pulmonary hypertension. Circulation.

[13] Ehlken N., Lichtblau M., Klose H. (2016). Exercise training improves peak oxygen consumption and haemodynamics in patients with severe pulmonary arterial hypertension and inoperable chronic thrombo-embolic pulmonary hypertension: a prospective, randomized, controlled trial. Eur Heart J.

[14] Buys R., Avila A., Cornelissen V.A. (2015). Exercise training improves physical fitness in patients with pulmonary arterial hypertension: a systematic review and meta-analysis of controlled trials. BMC Pulm Med.

[15] Yuan P., Yuan X.T., Sun X.Y., Pudasaini B., Liu J.M., Hu Q.H. (2015). Exercise training for pulmonary hypertension: a systematic review and meta-analysis. Int J Cardiol.

[16] Morris N.R., Kermeen F.D., Holland A.E. (2017). Exercise-based rehabilitation programmes for pulmonary hypertension. Cochrane Database Syst Rev.

[17] Grunig E., MacKenzie A., Peacock A.J. (2021). Standardized exercise training is feasible, safe, and effective in pulmonary arterial and chronic thromboembolic pulmonary hypertension: results from a large European multicentre randomized controlled trial. Eur Heart J.

[18] Naeije R., Richter M.J., Rubin L.J. (2022). The physiological basis of pulmonary arterial hypertension. Eur Respir J.

[19] Spruit M.A., Augustin I.M., Vanfleteren L.E. (2015). Differential response to pulmonary rehabilitation in COPD: multidimensional profiling. Eur Respir J.

[20] American Thoracic Society; American College of Chest Physicians (2003). ATS/ACCP statement on cardiopulmonary exercise testing. Am J Respir Crit Care Med.

[21] Davies L.C., Wensel R., Georgiadou P. (2005). Enhanced prognostic value from cardiopulmonary exercise testing in chronic heart failure by non-linear analysis: oxygen uptake efficiency slope. Eur Heart J.

[22] Rudski L.G., Lai W.W., Afilalo J. (2010). Guidelines for the echocardiographic assessment of the right heart in adults: a report from the American Society of Echocardiography endorsed by the European Association of Echocardiography, a registered branch of the European Society of Cardiology, and the Canadian Society of Echocardiography. J Am Soc Echocardiogr.

[23] Troosters T., Casaburi R., Gosselink R., Decramer M. (2005). Pulmonary rehabilitation in chronic obstructive pulmonary disease. Am J Respir Crit Care Med.

[24] Bennett D.A. (2001). How can I deal with missing data in my study?. Aust N Z J Public Health.

[25] Dwan K., Li T., Altman D.G., Elbourne D. (2019). CONSORT 2010 statement: extension to randomised crossover trials. BMJ.

[26] Grünig E., Lichtblau M., Ehlken N. (2012). Safety and efficacy of exercise training in various forms of pulmonary hypertension. Eur Respir J.

[27] Puente-Maestu L., Palange P., Casaburi R. (2016). Use of exercise testing in the evaluation of interventional efficacy: an official ERS statement. Eur Respir J.

[28] Ellis R., Shields N., Lim K., Dodd K.J. (2015). Eccentric exercise in adults with cardiorespiratory disease: a systematic review. Clin Rehabil.

[29] McHugh M.P. (2003). Recent advances in the understanding of the repeated bout effect: the protective effect against muscle damage from a single bout of eccentric exercise. Scand J Med Sci Sports.

[30] Ulrich S., Hasler E.D., Saxer S. (2017). Effect of breathing oxygen-enriched air on exercise performance in patients with precapillary pulmonary hypertension: randomized, sham-controlled cross-over trial. Eur Heart J.

[31] Weatherald J., Farina S., Bruno N., Laveneziana P. (2017). Cardiopulmonary exercise testing in pulmonary hypertension. Ann Am Thorac Soc.

[32] Sun X.-G., Hansen J.E., Garatachea N., Storer T.W., Wasserman K. (2002). Ventilatory efficiency during exercise in healthy subjects. Am J Respir Crit Care Med.

[33] Baba R. (2000). The oxygen uptake efficiency slope and its value in the assessment of cardiorespiratory functional reserve. Congest Heart Fail.

[34] Maenen M., Broek L.V.D., Somers L. (2022). Prediction of V’O2max in advanced COPD patients using the oxygen uptake efficiency slope. Eur Respir J.

[35] Nickel R., Troncoso F., Flores O. (2020). Physiological response to eccentric and concentric cycling in patients with chronic obstructive pulmonary disease. Appl Physiol Nutr Metab.

[36] Benza R.L., Miller D.P., Gomberg-Maitland M. (2010). Predicting survival in pulmonary arterial hypertension. Circulation.

[37] Lichtblau M., Bader P.R., Saxer S. (2020). Right atrial pressure during exercise predicts survival in patients with pulmonary hypertension. J Am Heart Assoc.

[38] Ho J.E., Zern E.K., Lau E.S. (2020). Exercise pulmonary hypertension predicts clinical outcomes in patients with dyspnea on effort. J Am Coll Cardiol.

[39] Tello K., Wan J., Dalmer A. (2019). Validation of the tricuspid annular plane systolic excursion/systolic pulmonary artery pressure ratio for the assessment of right ventricular-arterial coupling in severe pulmonary hypertension. Circ Cardiovasc Imaging.

[40] Naeije R., Manes A. (2014). The right ventricle in pulmonary arterial hypertension. Eur Respir Rev.

[41] Meyer K., Steiner R., Lastayo P. (2003). Eccentric exercise in coronary patients: central hemodynamic and metabolic responses. Med Sci Sports Exerc.

[42] Waller L., Krüger K., Conrad K., Weiss A., Alack K. (2020). Effects of different types of exercise training on pulmonary arterial hypertension: a systematic review. J Clin Med.

